# The Effects of SGLT2 Inhibitors on Lipid Profile and Kidney Function in Patients with Chronic Kidney Disease Regardless of Diabetes and Hypertension Status

**DOI:** 10.3390/metabo15040271

**Published:** 2025-04-13

**Authors:** Selena Gajić, Stefan Janković, Milorad Stojadinović, Kristina Filić, Ana Bontić, Jelena Pavlović, Ivana Mrđa, Kristina Petrović, Lara Hadži-Tanović, Jelena Žunić, Mihajlo Kostić, Aleksandra Kezić, Marko Baralić

**Affiliations:** 1 Clinic of Nephrology, University Clinical Center of Serbia, Pasterova 2, 11000 Belgrade, Serbia; 2 Faculty of Medicine, University of Belgrade, Dr Subotića Starijeg 8, 11000 Belgrade, Serbia

**Keywords:** chronic kidney disease, SGLT2 inhibitors, hypertension, diabetes mellitus type 2, triglycerides, cholesterol

## Abstract

Background: Chronic kidney disease (CKD) is a progressive, irreversible impairment of kidney function due to various etiologies. Numerous studies have shown that sodium-glucose cotransporter-2 inhibitors (SGLT2i) slow the progression of CKD, due to their pleiotropic effects. Therefore, there has been an increase in interest in their effects not only on kidney function but also on other parameters in patients with CKD. The aim of the study was to examine the effects of SGLT2i on serum lipid values and kidney function in patients with CKD undergoing SGLT2i treatment. Methods: This study was a retrospective data analysis of 75 patients with CKD on SGLT2i treatment. We compared the values of biochemical parameters, renal function outcomes, and blood pressure at two time points: baseline and 24 months after. Results: Total cholesterol (Chol) significantly decreased in all patients, while triglyceride (Tg) and low-density lipoprotein cholesterol (LDLc) levels also decreased in all patients. High-density lipoprotein cholesterol (HDLc) levels increased, but this increase was not significant. Creatinine clearance (Ccr) significantly decreased, and serum urea (Sur) significantly increased in all patients. The proteinuria (Prt) levels did not change significantly. The results showed that the diastolic blood pressure (DBP) significantly decreased in all patients. Conclusions: This study showed that the use of SGLT2i reduced total Chol in all patients with CKD during the 24-month follow-up, regardless of diabetes mellitus (DM) status. No significant differences were observed for the Tg, LDLc, and HDLc values.

## 1. Introduction

Chronic kidney disease (CKD) is defined as abnormalities in kidney structure and/or function that are present for a minimum of three months. It is characterized by a drop in the glomerular filtration rate (GFR) below 60 mL/min/1.73 m^2^ and/or the presence of one or more markers of kidney damage, including urine sediment abnormalities, albuminuria, persistent hematuria, electrolyte and other abnormalities due to tubular disorders, structural abnormalities detected by imaging, abnormalities detected by histology after kidney biopsy, and a history of kidney transplantation [1]. CKD can occur as a result of various etiological factors and most often occurs not only as a complication of uncontrolled arterial hypertension (HTN), diabetes mellitus type 1 or 2 (T1DM or T2DM), glomerulonephritis (GN), autosomal dominant polycystic kidney disease (ADPKD), but also tubulointerstitial nephritis (TIN), and other causes [2,3]. Bearing in mind that approximately 10% of the world’s adult population has some degree of CKD, which is responsible for 1–2 million deaths per year; therefore, CKD is considered to have a pandemic character [2,4]. The burden of CKD is on the rise worldwide due to the increase in life expectancy and the greater prevalence of obesity, HTN, and DM [5,6]. It is predicted that by 2040, it will be the world’s fifth-leading cause of death [2,4]. Therefore, it is necessary to develop and use new therapeutic modalities that will slow down the progression of the disease and preserve kidney function to prevent the development of end-stage kidney disease (ESKD) and the need for treatment with some of the modalities of kidney replacement therapy (KRT) [7].

Sodium-glucose cotransporter-2 inhibitors (SGLT2i) were developed as glucose-lowering drugs for the treatment of diabetes [8,9]. SGLT2i blocks glucose (Gly) reabsorption in the proximal renal tubules [10] and, at the same time, sodium reabsorption, ultimately achieving glycosuria (and consequently, regulation of fasting and postprandial glycemia), natriuresis, and osmotic diuresis. It should be noted that the amount of Gly eliminated by this mechanism depends on the concentration of Gly in the blood and GFR. Furthermore, increased delivery of sodium to the distal tubule increases tubuloglomerular feedback and reduces intraglomerular pressure, which, in combination with osmotic diuresis, consequently leads to a decrease in preload and afterload and thus, among other things, has beneficial effects on cardiac remodeling and preservation of kidney function [11,12]. Several clinical studies have demonstrated their positive effects in reducing cardiovascular (CV) morbidity and mortality [13,14,15]. In real-world studies, SGLT2i is now recognized as a crucial therapy for slowing CKD progression and a cornerstone therapy for both heart failure with reduced ejection fraction (HFrEF) and HF with preserved EF (HFpEF) [16,17]. Consequently, these medications are frequently prescribed for individuals with CKD [1,18,19] and HF [16,17].

Given that dyslipidemia is a common factor in CKD and CV disease (CVD), which complicates the illness and its progression, the treatment of dyslipidemia would significantly contribute to slowing the progression of both diseases. In clinical trials, it has been shown that the reduction in low-density lipoprotein cholesterol (LDLc) levels in patients with CKD is directly proportional to the decrease in CVD risk [20]. Therefore, investigating the effect of SGLT2i on lipid and lipoprotein levels is important to elucidate their potential role in lowering CV risk observed with SGLT2i treatment. However, there is a lack of focus on the systematic measurement and reporting of lipid and lipoprotein levels in randomized placebo-controlled trials of SGLT2i. Thus, it is not clear whether SGLT2i affects lipid and lipoprotein levels in a clinically relevant manner and whether the effect varies with SGLT2i dose or type [8]. This study aimed to examine the effects of SGLT2i on serum lipid values and kidney function in 24-month-treated patients with CKD.

## 2. Materials and Methods

### 2.1. Study Participants

This retrospective cohort study used data from the University Clinical Center of Serbia (UCCS) information system. The study cohort included 75 patients aged ≥18 years with newly diagnosed CKD stage 1–4 who had an indication for starting SGLT2i therapy. All patients were treated with SGLT2i, dapagliflozin 10 mg orally once daily for 24 months, from October 2022 to October 2024, as outpatients at the UCCS Clinic of Nephrology. We defined CKD and CKD stages according to the Kidney Disease: Improving Global Outcomes (KDIGO) guidelines [1]. All patients underwent follow-up visits every 3–6 months, depending on the CKD stage, by the same nephrologist. The Ethics Committee of UCCS granted approval to collect the medical data and carry out the study (Decision Number 1322/X-7, dated 10 October 2022). This study was conducted following the Declaration of Helsinki. Demographic, anamnestic, and laboratory data were extracted from the medical history data of the UCCS information system. All patients were on a hygienic-dietary regimen, 72% of patients were on maximally tolerated doses of renin-angiotensin-aldosterone system inhibitors (RAASi), and 41% of patients were on lipid-lowering therapy.

### 2.2. Laboratory Tests

Blood and urine samples and blood pressure measurements were obtained from all patients at each nephrology visit, and they were compared for this study at two time points: at the start of SGLT2i treatment and 24 months after. All biochemical samples were collected early in the morning, 12 h after the patient’s last meal. For biochemical analyses, blood was collected in tubes without the addition of anticoagulants. Blood samples were collected from all patients in the central laboratory of the UCCS without storage but were immediately processed. Biochemical parameters, such as serum Gly, serum urea (Sur), serum creatinine (Scr), creatinine clearance (Ccr), glycated hemoglobin (HbA1c), triglycerides (Tg), total cholesterol (Chol), high-density lipoprotein cholesterol (HDLc) and LDLc, and proteinuria (Prt) were determined using routine laboratory test procedures on an automated analyzer Architect ci8200 (Abbott Diagnostics, Wiesbaden, Germany).

### 2.3. Statistical Analysis

The normal distribution of continuous variables was tested using the Kolmogorov–Smirnov test. Continuous variables with a normal distribution are presented as mean ± standard deviation (SD), and continuous variables that did not show a normal distribution are presented as median values and interquartile ranges (IQR). Categorical variables are presented as counts and percentages. The differences between the two measurements (baseline vs. after 24 months) were evaluated using the paired samples *t*-test or its non-parametric equivalent, the Wilcoxon signed-rank test, depending on the data distribution. The Mann–Whitney U test was used to compare renal outcomes (Ccr reduction, estimated GFR (eGFR) reduction, and Prt reduction) in the groups with and without DM and with and without HTN. Multiple linear regression was used to assess the impact of both independent and confounding variables on the Prt reduction. Statistical analyses were performed using the Statistical Package for Social Sciences, version 18 (SPSS, Chicago, IL, USA). Statistical significance was set at *p* < 0.05.

## 3. Results

Seventy-five patients were included in the study, of which 43 were female (57.3%) and 32 were male (42.7%). The average age of the patients was 62 ± 15.39 years old. 33 (44%) patients were on angiotensin-converting enzyme inhibitor (ACEi) therapy, while 21 (28%) were on angiotensin II receptor blockers (ARBs) therapy. More than half of the cohort did not receive lipid-lowering therapy (58.7%), 28 (37.3%) patients were receiving only statins, two (2.7%) patients were on statin and fibrate therapy, and one patient was on a statin and ezetimibe therapy. 34 (45.3%) patients had T2DM, and 64 (85.3%) had HTN. The leading cause of CKD in the cohort was DM (45.4%), followed by HTN in 27 (36.0%) patients, GN in 13 (17.3%) patients, and TIN in one patient (1.3%). Most patients had CKD stage 3 (54.6%), 17 (22.7%) patients had CKD stage 1 or 2, and 17 (22.7%) had CKD stage 4 (Table 1).

In the study, we compared the values of biochemical parameters (Gly, Sur, Scr, HbA1c, Chol, HDLc, LDLc, and Tg) at two times, at the beginning of the study, which was defined as a baseline measure and 24 months after SGLT2i therapy was started. The results showed that Sur significantly increased (*p* = 0.032) and total Chol significantly decreased (*p* < 0.001) after 24 months of follow-up. The values of the other measured biochemical parameters did not change significantly (Table 2). Other renal function parameters were also compared in the study: Ccr, eGFR, and Prt. The results showed a statistically significant decrease in Ccr (*p* = 0.021) and a decrease in Prt, but the difference was not significant (*p* = 0.232). The eGFR did not change significantly after 24 months of follow-up (*p* = 0.360) (Table 2). The results showed that SGLT2i therapy led to a significant decrease in diastolic blood pressure (DPB) (*p* = 0.019). The systolic blood pressure (SBP) values did not change significantly (*p* = 0.087) (Table 2).

The values of the measured biochemical and renal function parameters did not change significantly, regardless of DM status, except for LDLc, which significantly decreased in patients with DM (*p* = 0.005). In patients without DM, LDLc also decreased, but the difference was not significant (*p* = 0.207) (Table 3). SBP and DBP significantly decreased in patients with DM (*p* = 0.016 and 0.013, respectively) but did not change significantly in patients without DM (*p* = 0.965 and 0.404, respectively) (Table 3).

Sur significantly increased in patients with HTN (*p* = 0.017) and decreased in patients without HTN, but the difference was not significant (*p* = 0.594). Total Chol and LDLc levels significantly decreased in patients with HTN (*p* < 0.001 and *p* = 0.041, respectively) and in patients without HTN, but the difference was not significant (*p* = 0.472 and 0.965, respectively) (Table 4).

Renal function outcomes (Ccr reduction, eGFR reduction, and Prt reduction) did not change significantly after 24 months of follow-up, regardless of both DM and HTN status (Table 5 and Table 6).

The final multiple linear regression model of 24 months proteinuria reduction predictors included the following variables: age, sex, primary kidney disease, use of RAASi, SBP and DBP, baseline eGFR (eGFRb), baseline Chol, baseline Tg, and baseline 24-h proteinuria (Prtb). All assumptions for multiple linear regression were satisfied, including independence of observations, normality of residuals, absence of multicollinearity, linear relationships between predictors and the dependent variable, and homoscedasticity. One outlier was excluded from the final analysis. The regression model was statistically significant (F = 8.967, *p* < 0.001) and explained 56.6% of the variability in dependent variables (R² = 0.566). The significant predictors of the 24 months proteinuria reduction were Prtb and eGFRb, and age was a marginally significant predictor (Table 7).

No complicated urinary infections were recorded during the monitoring period. One patient had diarrheal disease. No hospitalization was registered due to heart failure or any amputation of the extremities. No lethal outcomes were observed during the follow-up period.

## 4. Discussion

CKD is a severe and significant public health issue worldwide [4]. Given that the disease is progressive and irreversible, modern nephroprotective therapy aims to slow down the further progression of the disease and to delay the development of ESKD, and thus the application of KRT, such as hemodialysis, peritoneal dialysis, or kidney transplantation [21]. Since the advent of ACEi, SGLT2i has been the most significant group of drugs considered to slow the progression of CKD [22]. By blocking the SGLT2 receptor, these drugs increase natriuresis and glycosuria [23]. Increased natriuresis is registered by the cells of the macula densa, which respond by increasing adenosine triphosphate (ATP) production, which, either directly or via adenosine, leads to vasodilatation of the afferent arteriole, which reduces intraglomerular pressure and glomerular hyperfiltration, and thus reducing Prt and the progression of kidney damage [24,25]. In contrast to large clinical trials [26,27] and meta-analyses of SGLT2i effects in CKD patients [28], in which SGLT2i treatment reduced the risk of kidney disease progression, in our cohort, Prt did not change significantly, Sur increased, and Ccr decreased significantly. Our cohort was similar in age to the DAPA-CKD cohort, but we had a higher percentage of female participants and a higher percentage of participants with DM. In the DAPA-CKD cohort, more than 98% of the participants were on RAASi therapy, while in our cohort, 72% of the participants were. In addition, in the DAPA-CKD cohort, the mean eGFR was higher than that in our cohort, but we had a higher percentage of patients with CKD stage 4. In other studies from the Serbian population, like the study by Ristanović et al. [29], no significant variations were observed in eGFR after one year of therapy with SGLT2i in CKD patients; however, in contrast to our study, in their cohort, Prt was significantly reduced. Their participants were younger than ours, and they had more participants with CKD stage 2 and fewer participants with CKD stage 4 than our cohort. In our study, after subgroup analysis according to DM and HTN status, the results showed that Prt was reduced in both diabetic and non-diabetic groups, but the reduction was not significant. CCr was reduced in the non-diabetic group and increased in the diabetic group, but the changes were not significant. According to HTN status, Ccr was reduced in both groups but without statistical significance. Prt was reduced in the HTN group and increased in the non-HTN group, but the results were insignificant. There is a lack of studies on Prt and Ccr changes in CKD patients with SGLT2i treatment according to DM and HTN status in the Serbian population for comparison with our results.

Gly and sodium, as osmotically active particles, draw water with them and lead to a decrease in the volume of circulating fluid and, therefore, to a decrease in BP [23], which is very significant if we consider that, according to the literature, HTN is the second most common cause of CKD [30]. Unlike our study, which showed that SGLT2i statistically significantly reduced only DBP, a meta-analysis by Zhang et al. showed that SGLT2i led to a reduction in both SBP and DBP [31]. Reduction of both SBP and DBP in our study was registered in the diabetic group but not in the non-diabetic group.

SGLT2 inhibitors lower glycemia by preventing Gly reabsorption. Hence, they are used in patients with T2DM [32], which is the leading cause of CKD [2]. In our study, Gly and HbA1c levels were reduced after a 24-month follow-up, but the difference was not statistically significant. Numerous studies have shown that, in addition to lowering glycemia, SGLT2i reduces the frequency of diabetic complications, the need for hospitalization due to HF, the progression of CKD, and amputations [27,33]. Our study did not have data for HF hospitalizations, but we did not register amputations or deaths during the 24-month follow-up.

In addition, SGLT2i, by changing the expression of various adhesive molecules and protecting against the harmful effects of reactive oxygen species and proinflammatory cytokines, prevents the occurrence of podocytopathies and the multiplication of the mesangial matrix, thereby preventing the progression of glomerular damage in GN [34,35], which is the third leading cause of CKD [36]. Previous studies have shown that in certain GN, SGLT2i reduced Prt [37]. In our study, Prt did not change significantly, but we had only 13 patients with GN in the cohort. A pre-specified analysis of the DAPA-CKD trial indicated a kidney-protective effect of SGLT2i in immunoglobulin A (IgA) nephropathy, and it is now a recommended therapy. SGLT2i was previously suggested as a potential treatment option for focal segmental glomerulosclerosis (FSGS); however, in a more recent short-term crossover randomized controlled trial (DIAMOND), patients with FSGS treated with dapagliflozin had a nonsignificant reduction in Prt compared to the placebo group. In a pre-specified analysis of the DAPA-CKD trial in patients with FSGS, urine albumin-to-creatinine ratio levels were similar between the dapagliflozin and placebo groups at 12 months; therefore, dedicated multicenter randomized controlled trials of the different forms of FSGS are needed to address the efficacy of SGLT2i in this heterogeneous condition [38].

Our research supports the hypothesis, discussed in many studies, that there are several assumptions about how SGLT2i may affect lipid metabolism. A lower blood Gly level reduces insulin production and increases serum glucagon levels, which is especially pronounced in patients with T2DM who already have a relative lack of insulin [39]. Glucagon, in addition to its primary effect of increasing serum Gly, lowers Chol, stimulates lipolysis and lowers Tg levels [40,41]. According to a review article by Chilton [42], a slight increase in LDLc and HDLc and a decrease in Tg levels were observed in patients treated with SGLT2i. Our study showed a significant decrease in total Chol levels in all patients, as well as a decrease in Tg and LDLc levels and an increase in HDLc levels; however, these results were not significant. Other authors, such as Tentolouris A. et al. [43], showed that SGLT2i treatment led to a small but statistically significant increase in Chol levels and a decrease in Tg levels. A meta-analysis by Szekeres et al. explained the CV and kidney-protective effects of SGLT2i despite an increase in LDLc levels, which was observed in several clinical studies [13].

Nevertheless, this increase in LDLc occurs in the setting of other beneficial changes in plasma lipoprotein metabolism. SGLT2i affects lipid metabolism at several different levels. They decrease lipid accumulation in visceral fat, regulate serum lipoprotein levels, beneficially change the ratio of LDL particles, reduce lipid oxidation, and shift substrate utilization toward the use of ketone bodies, which are more efficient in myocardial metabolism and less reactive oxygen species are created through their oxidation, affecting β-oxidation and the transportation of lipid molecules in the cells. These favorable changes in lipid metabolism may counteract the net increase in LDL levels [13]. In our study, after subgroup analysis according to DM and HTN status, significance was shown in the reduction of LDLc values in groups of patients with DM and HTN compared with groups of patients without DM and HTN, in which LDLc values decreased but not significantly.

Metabolic dysfunction-associated steatotic liver disease (MASLD) is a leading cause of cirrhosis and is associated with hyperlipidemia. Today, an increasing number of researchers are advocating the use of SGLT2i in the treatment of MASLD because possible therapeutic interventions that improve cardiometabolic risk factors may be useful for improving MASLD. The effects of such therapeutic interventions on the accumulation of lipids, lipoproteins, and apoproteins in the liver and on hepatic steatosis and fibrosis remain unclear; however, it can be assumed that the effects of SGLT2i are multifaceted and beneficial to different systems with metabolic functions [44].

This study had several limitations. First, its retrospective design may have resulted in missing or incomplete information and is susceptible to selection and recall biases. Second, the study was conducted at a single center with a relatively small sample size (75 patients), which may limit the generalizability of the findings and statistical power to detect subtle differences. However, all patients within the first month of receiving indications for SGLT2i treatment of CKD were included. Third, although multiple linear regression was used to adjust for confounding variables, the relatively low R-squared value may affect the reliability of the conclusions.

## 5. Conclusions

This study showed that the use of SGLT2i affects the reduction of total Chol in all CKD patients during the 24-month follow-up, regardless of DM status. No significance was shown for the Tg, LDLc, and HDLc values. The reduction in LDLc values was significant in patients with DM and HTN compared with those without DM and HTN, in which LDLc values decreased but not significantly. There was a significant decrease in SBP and DBP in the DM group and better control of DBP in the group of patients with HTN. Multiple linear regression analysis showed that Prtb and eGFRb were significant predictors, and age was a marginally significant predictor of Prt reduction at 24 months. SGLT2i can be considered a safe group of medicines in terms of urinary infections, which have not been reported in patients with diabetes.

## Figures and Tables

**Table 1:** Baseline demographic data of the patients.

	Overall, N = 75
Age-years, mean ± SD	61.96 ± 15.39
Sex-male, *n* (%)	32 (42.7%)
T2DM, *n* (%)	34 (45.3%)
HTN, *n* (%)	64 (85.3%)
ACEi, *n* (%)	33 (44.0%)
ARBs, *n* (%)	21 (28.0%)
**Causes of CKD, *n* (%)**	
DM	34 (45.4%)
HTN	27 (36.0%)
GN	13 (17.3%)
TIN	1 (1.3%)
**CKD stage, *n* (%)**	
CKD stage 1–2	17 (22.7%)
CKD stage 3	41 (54.6%)
CKD stage 4	17 (22.7%)
**Lipid-Lowering Therapy**	
Non	44 (58.7%)
Only statin	28 (37.3%)
Statin and fibrate	2 (2.70%)
Statin and ezetimibe	1 (1.30%)

Data are presented as mean ± SD or number (percentage). ACEi: Angiotensin-converting enzyme inhibitors; ARBs: Angiotensin II receptor blockers; CKD: Chronic kidney disease; T2DM: Diabetes mellitus type 2; DM: Diabetes mellitus; HTN: Hypertension; GN: Glomerulonephritis; TIN: Tubulointerstitial nephritis.

**Table 2:** Biochemical parameters in two measurements in patients with CKD: Baseline—values at the beginning of the study, 24 months—values obtained after 24 months of SGLT2i treatment. ^1^ Paired-sample *t*-test or Wilcoxon signed-rank test.

Parameters	Baseline	24 Months	*p*-Value ^1^
Gly, (mmol/L), median (IQR)	6.10 (2.00)	5.80 (1.40)	0.592
Sur, (mmol/L), median (IQR)	8.40 (5.60)	8.75 (6.90)	**0.032**
Scr, (umol/L), median (IQR)	125.00 (74.00)	134.00 (72.00)	0.068
Ccr, (mL/min), median (IQR)	51.30 (31.80)	47.85 (31.70)	**0.021**
eGFR, (mL/min), median (IQR)	41.00 (28)	41.00 (23)	0.360
HbA1c, (%), median (IQR)	6.30 (2.00)	6.20 (2.00)	0.889
Chol, (mmol/L), mean ± SD	5.28 ± 1.28	4.98 ± 1.19	**<0.001**
HDLc (mmol/L), median (IQR)	1.24 (0.60)	1.30 (0.60)	0.615
LDLc (mmol/L), mean ± SD	3.17 ± 1.10	2.53 ± 0.99	0.060
Tg, (mmol/L), median (IQR)	1.68 (1.10)	1.37 (1.01)	0.137
SBP, (mmHg), median (IQR)	120.00 (30.00)	120.00 (15.00)	0.087
DBP, (mmHg), median (IQR)	80.00 (10.00)	70.00 (10.00)	**0.019**
Prt, (g/24 h), median (IQR)	0.20 (1.00)	0.20 (0.70)	0.232
Ccr reduction, (mL/min), mean ± SD	-	2.04 ± 17.52	-
eGFR reduction, (mL/min), median (IQR)	-	0.00 (11.00)	-
Prt reduction, (g/24 h), median (IQR)	-	0.02 (0.32)	-

Gly: Glucose; Sur: Serum urea; Scr: Serum creatinine; Prt: Proteinuria; Ccr: Creatine clearance; HbA1c: Glycated hemoglobin; Chol: cholesterol; HDLc: High-density lipoprotein cholesterol; LDLc: Low-density lipoprotein cholesterol; Tg: Triglyceride; SBP: Systolic blood pressure; DBP: Diastolic blood pressure; eGFR: estimated glomerular filtration rate; *p* baseline measure vs. measure after 24 months of SGLT2i treatment. Significance is indicated in bold. SD: standard deviation; IQR: interquartile range.

**Table 3:** Biochemical parameters at two time points in CKD patients with or without DM: Baseline—values at the beginning of the study, 24 months—values obtained after 24 months of SGLT2i treatment. ^1^ Paired-sample *t*-test or Wilcoxon signed-rank test.

Parameters	DM	Baseline	24 Months	*p*-Value ^1^
Gly, (mmol/L), mean ± SD, median (IQR)	No	5.45 ± 0.93	5.41 ± 0.74	0.898
	Yes	7.40 (3.75)	6.40 (2.60)	0.416
Sur, (mmol/L), median (IQR)	No	6.50 (3.40)	6.90 (4.70)	0.267
	Yes	11.15 (5.20)	11.70 (7.90)	0.067
Scr, (µmol/L), median (IQR)	No	110.00 (50.00)	122.00 (48.00)	0.147
	Yes	164.00 (91.00)	163.50 (129.00)	0.237
Ccr, (mL/min), median (IQR)	No	60.00 (36.40)	48.90 (35.00)	0.181
	Yes	41.80 (21.00)	45.00 (29.70)	0.641
eGFR, (mL/min), median (IQR)	No	47.00 (25.00)	45.00 (21.00)	0.072
	Yes	34.50 (18.00)	35.00 (22.00)	0.673
Chol, (mmol/L), median (IQR)	No	5.30 (1.72)	5.00 (1.47)	0.131
	Yes	5.06 (1.90)	4.43 (1.90)	0.150
HDLc, (mmol/L), mean ± SD	No	1.44 ± 0.45	1.39 ± 0.33	0.265
	Yes	1.18 ± 0.30	1.17 ± 0.31	0.953
LDLc, (mmol/L), mean ± SD	No	3.44 ± 1.14	2.78 ± 1.22	0.207
	Yes	2.81 ± 0.95	2.33 ± 0.76	**0.005**
Tg, (mmol/L), median (IQR)	No	1.60 (1.23)	1.35 (0.99)	0.779
	Yes	1.81 (1.23)	1.52 (1.27)	0.320
SBP, (mmHg), median (IQR)	No	120.00 (20.00)	120.00 (15.00)	0.965
	Yes	125.00 (31.00)	120.00 (26.00)	**0.016**
DBP, (mmHg), median (IQR)	No	80.00 (10.00)	70.00 (10.00)	0.404
	Yes	80.00 (10.00)	70.00 (10.00)	**0.013**
Prt, (g/24 h), median (IQR)	No	0.28 (0.99)	0.23 (0.70)	0.161
	Yes	0.20 (0.90)	0.19 (0.81)	0.845

Gly: Glucose; Sur: Serum urea; Scr: Serum creatinine; Prt: Proteinuria; Ccr: Creatine clearance; Chol: Cholesterol; HDL: High-density lipoprotein cholesterol; LDL: Low-density lipoprotein cholesterol; Tg: Triglyceride; SBP: Systolic blood pressure; DBP: Diastolic blood pressure; eGFR: estimated glomerular filtration rate; DM: Diabetes mellitus; SD: standard deviation; IQR: interquartile range; *p* baseline measure vs. measure after 24 months of SGLT2i treatment. Significance is in bold.

**Table 4:** Biochemical parameters at two time points in patients with CKD with and without HTN: Baseline—values at the beginning of the study, 24 months—values obtained after 24 months of SGLT2i treatment. ^1^ Paired-sample *t*-test or Wilcoxon signed-rank test.

Parameters	HTN	Baseline	24 Months	*p*-Value ^1^
Gly, (mmol/L), median (IQR)	No	6.20 (2.50)	6.40 (4.40)	0.068
	Yes	6.05 (1.97)	5.70 (1.30)	0.139
Sur, (mmol/L), median (IQR)	No	9.10 (5.70)	7.90 (6.70)	0.594
	Yes	8.35 (5.70)	9.30 (7.20)	**0.017**
Scr, (µmol/L), median (IQR)	No	135.00 (57)	132.50 (63)	0.683
	Yes	124.00 (79)	138.00 (72)	0.074
Chol, (mmol/L), mean ± SD	No	5.68 ± 1.28	4.91 ± 0.91	0.472
	Yes	5.21 ± 1.28	5.00 ± 1.24	**<0.001**
Tg, (mmol/L), median (IQR)	No	2.27 (2.23)	1.48 (1.54)	0.445
	Yes	1.63 (1.10)	1.31 (0.96)	0.172
HDLc, (mmol/L), median (IQR)	No	1.22 (0.50)	1.29 (0.80)	0.715
	Yes	1.24 (0.60)	1.30 (0.50)	0.460
LDLc, (mmol/L), mean ± SD	No	3.17 ± 1.14	2.78 ± 0.92	0.965
	Yes	3.17 ± 1.11	2.46 ± 1.03	**0.041**
SBP, (mmHg), median (IQR)	No	120.00 (10)	115.00 (15)	0.886
	Yes	120.00 (30)	120.00 (24)	0.070
DBP, (mmHg), median (IQR)	No	70.00 (10)	70.00 (10)	0.147
	Yes	80.00 (10)	70.00 (10)	0.060
Prt, (g/24 h), median (IQR)	No	0.11 (0.20)	0.23 (0.19)	0.176
	Yes	0.26 (0.98)	0.20 (0.72)	0.103
Ccr, (mL/min), median (IQR)	No	48.80 (67.20)	43.50 (79.60)	0.779
	Yes	51.80 (31.40)	47.85 (24.80)	0.283
eGFR, (mL/min), median (IQR)	No	43.00 (28)	42.00 (26)	0.993
	Yes	40.50 (27)	41.00 (23)	0.365

Gly: Glucose; Sur: Serum urea; Scr: Serum creatinine; Prt: Proteinuria; Ccr: Creatine clearance; Chol: Cholesterol; HDL: High-density lipoprotein cholesterol; LDL: Low-density lipoprotein cholesterol; Tg: Triglyceride; SBP: Systolic blood pressure; DBP: Diastolic blood pressure; eGFR: estimated glomerular filtration rate; HTN: Hypertension; SD: standard deviation; IQR: interquartile range; *p* baseline measure vs. measure after 24 months of SGLT2i treatment. Significance is in bold.

**Table 5:** Renal outcomes in patients with and without DM: A comparative analysis (Mann–Whitney U test).

Parameters	DM	Median (IQR)	*p*-Value
Ccr reduction, (mL/min)	No	2.70 (20.60)	0.620
	Yes	0.75 (18.83)	
eGFR reduction, (mL/min)	No	0.00 (11.00)	0.307
	Yes	0.00 (9.00)	
Prt reduction, (g/24 h)	No	0.04 (0.37)	0.397
	Yes	0.01 (0.22)	

Ccr: Creatine clearance; eGFR: estimated glomerular filtration rate; Prt: Proteinuria; DM: Diabetes mellitus; IQR: interquartile range.

**Table 6:** Renal outcomes in patients with and without HTN: A comparative analysis (Mann–Whitney U test).

Parameters	HTN	Median (IQR)	*p*-Value
Ccr reduction, (mL/min)	No	−1.25 (13.50)	0.971
	Yes	2.70 (20.75)	
eGFR reduction, (mL/min)	No	0.00 (4.00)	0.878
	Yes	0.00 (11.00)	
Prt reduction, (g/24 h)	No	−0.03 (0.15)	0.067
	Yes	0.06 (0.35)	

Ccr: Creatine clearance; eGFR: estimated glomerular filtration rate; Prt: Proteinuria; HTN: Hypertension; IQR: interquartile range.

**Table 7:** Significant and marginally significant predictors of the 24 months proteinuria reduction. Multiple linear regression analysis.

Predictor	B	β (95% CI)	*p*-Value
Age	0.016	0.221 (0.000–0.033)	**0.053**
Prtb	0.604	0.749 (0.435–0.773)	**<0.001**
eGFRb	0.021	0.240 (0.002–0.039)	**0.028**

Prtb: baseline 24-h proteinuria; eGFRb: baseline estimated glomerular filtration rate; β = Standardized regression coefficient; B = Unstandardized regression coefficient; CI = Confidence Interval; The model constant (intercept) = −2.269, marginally significant (*p* = 0.053).

## Data Availability

The data presented in this study are available upon request from the corresponding author. The data are not publicly available due to privacy of patients.

## Abbreviations

The following abbreviations are used in this manuscript:

CKD	Chronic kidney disease
SGLT2i	Sodium-glucose cotransporter-2 inhibitors
eGFR	Estimated glomerular filtration rate
HTN	Hypertension
T1DM	Diabetes mellitus type 1
T2DM	Diabetes mellitus type 2
GN	Glomerulonephritis
ADPKD	Autosomal dominant polycystic kidney disease
TIN	Tubulointerstitial nephritis
LDLc	Low-density lipoproteins cholesterol
HDLc	High-density lipoprotein cholesterol
UCCS	University Clinical Center of Serbia
Ccr	Creatinine clearance
Prt	Proteinuria
SD	Standard deviation
IQR	Interquartile range
ACEi	Angiotensin-converting enzyme inhibitors
ARBs	Angiotensin II receptor blockers
ESKD	End-stage kidney disease
KRT	Kidney replacement therapy
ATP	Adenosine triphosphate
FSGS	Focal segmental glomerulosclerosis
RAASi	Renin-angiotensin-aldosterone system inhibitors
CI	Coinfidence Interval
B	Unstandardized regression coefficient
β	Standardized regression coefficient
Gly	Glucose
Sur	Serum urea
Scr	Serum creatinine
HbA1c	Glycated hemoglobin
Tg	Triglyceride
Chol	Cholesterol
SBP	Systolic blood pressure
DBP	Diastolic blood pressure
Prtb	Baseline proteinuria
eGFRb	Baseline estimated glomerular filtration rate
CVD	Cardiovascular disease
RAASi	Renin-angiotensin-aldosterone system inhibitors
KDIGO	Kidney Disease: Improving Global Outcomes
HFrEF	Heart failure with reduced ejection fraction
HFpEF	Heart failure with preserved ejection fraction
MASLD	Metabolic dysfunction-associated steatotic liver disease
